# Sources of the Deposition of Submicron Soot Particles on Plant Leaves

**DOI:** 10.3390/biology14060583

**Published:** 2025-05-22

**Authors:** Qingyang Liu

**Affiliations:** College of Ecology and Environment, Nanjing Forestry University, Nanjing 210037, China; qyliu@njfu.edu.cn

**Keywords:** dry deposition, trees, soot particles, sources

## Abstract

Submicron soot particles (with an aerodynamic diameter of less than 1.0 μm) are found to be one of the major factors resulting in global warming and health burdens. However, research on the biomonitoring of submicron soot particles and their associated sources using tree leaves has not been comprehensively conducted. This study investigated the seasonal trends of submicron soot particles on the leaves of seven tree types collected from four individual seasons across two years in Nanjing, in the Yangtze River Delta region of China, and performed source apportionment using stable carbon isotope analysis. Significant seasonal variations in submicron soot particles were observed on tree leaves of seven tree types, with average levels of 0.3 to 0.5 mg m^−2^ during summer and 0.5 to 1.3 mg m^−2^ during winter. The levels of submicron soot particles varied significantly across various tree types. In contrast, the levels of δ^13^C were not found to change significantly across different types. The levels of δ^13^C ranged from −26.3‰ to −20.9‰ in winter and from −24.0‰ to −18.1‰ in summer, with fossil fuels accounting for 56% and 78% of submicron soot in winter and summer on average, respectively. These results demonstrate that tree leaves can serve as a low-cost and effective biomonitoring tool for assessing the source status of submicron soot.

## Simple Summary

Submicron soot particles (smaller than 1.0 μm) contribute to global warming and health risks. This study analyzed soot levels on leaves from seven tree types in Nanjing, China, across four seasons over two years. The results showed higher soot levels in winter (0.5–1.3 mg/m^2^) compared to summer (0.3–0.5 mg/m^2^), with variations among tree species. Stable carbon isotope analysis revealed that fossil fuels were the primary source, contributing 56% in winter and 78% in summer. The findings suggest tree leaves can be a cost-effective tool for monitoring submicron soot pollution and its sources.

## 1. Introduction

Atmospheric soot particle emissions are found to be associated with a warming climate and deteriorating air quality [1,2,3]. Soot particles affect the climate system in four main ways, i.e., direct effects, indirect effects, semi-direct effects, and indirect surface albedo effects [4,5,6]. As an important component of atmospheric aerosol, soot particles can absorb and scatter solar short-wave radiation through direct impacts to heat the lower atmosphere, resulting in positive radiative forcing at the top of the atmosphere and negative radiative forcing at the surface [6,7]. This role can affect the radiative balance of the terrestrial air system, thus causing global or regional climate warming [3]. Nucleated soot particles can change the microphysical processes of clouds [8]. The increase of cloud condensation nuclei leads to the enhancement of cloud droplet number density, the decrease of cloud droplet finite radius, the growth in cloud albedo and optical thickness, and negative radiative forcing on the atmosphere [9]. In addition, atmospheric negative radiative forcing is caused by the increase of cloud droplet density and the decrease of the effective radius, which inhibits warm cloud precipitation and leads to the rise of cloud lifetimes or thickness [9,10]. Apart from the heating of the atmosphere by the absorption of solar radiation by soot particles, the soot particles can promote the evaporation of cloud droplets and the reduction of cloud cover, resulting in local warming of the atmosphere and changes in cloud cover and water vapor channels [11,12]. Soot particles settling on the surface can also affect the climate by changing the albedo of the underlying surface [6]. On the cryosphere or sea ice, soot particles on the surface of snow and ice reduce the albedo of the snow-covered area and enhance the absorption of solar radiation by the snow, thus accelerating the melting of the snow [2,10].

Soot particles are aggregates, which are composed of many carbon nanospheres produced by incomplete combustion of fossil fuels and biomass fuels [12,13,14]. The physical and chemical processes of soot formation are very complex and affected by various factors, such as the type of fuel, the mixtures of fuel in the combustion equipment, the flow, and the residence temperature and time [15]. The formation of soot particles includes the following processes, i.e., the formation of soot precursors, soot particle nucleation, the surface growth of soot particles, soot particles colliding and aggregating, and soot oxidation [16,17]. Atmospheric soot could be transported over a long distance from source sectors along with atmospheric aerosols [18]. Prior studies have shown that the amounts of transported soot through the atmosphere are estimated to be from approximately 6 to 28 million tons per year [19]. As a result, soot particles are widely distributed in the global environment, even in far remote regions such as Antarctic glaciers and Tibet [20,21]. Soot particles come from a wide range of sources, with more than 64 emission sources [22]. There are five major emission sources, which include diesel engines, coal burning, residential solid fuel combustion, open biomass burning, and other emission sources (e.g., gasoline engines [22]). It is estimated that more than 90% of the total global soot emissions are from diesel engines, coal burning, residential solid fuel combustion, and open biomass burning [23,24]. Diesel engine emissions dominated the total global soot emissions, which account for about 20% of total emissions [8,14]. According to a study in China, the total amount of soot emission in China is approximately 1.88 million tons [25,26]. The largest source contributing to the total emissions is coal combustion (54%), followed by biomass combustion (32%). In comparison, natural sources (e.g., forest and grassland fires) emit trace amounts, accounting for 0.1% of the total emissions [25].

Stable carbon isotopes (δ^13^C) can be used to trace the source of soot and estimate the contribution from diverse sources, because there are certain differences in the values of soot from different sources [27,28]. The values of δ^13^C for soot aerosol emission sources are found to be ranging from −25‰ to −24‰ for diesel vehicles, −22‰ to −18‰ for marine sources, −23‰ to −21‰ for coal combustion, −29‰ to −24‰ for coal combustion, −34‰ to −25‰ for C3 plant combustion, and −13‰ for C4 plant combustion [29,30,31]. However, it is worth noting that the δ^13^C values of emission sources exhibit strong regional characteristics [28]. For example, studies have shown that the δ^13^C values of C3 plants in the United States, Japan, and Australia are distinct, which varied from −28‰ to −34.7‰, from −24.6‰ to −29.2‰, and from −26.1‰ to −29.7‰, respectively [28,32,33]. Stable carbon isotopes could be used to estimate the source contribution of soot. Liu et al. [34] analyzed δ^13^C values of carbonaceous aerosols from two rural villages in Fenwei Plain in China and proved that solid fuels are an important source of carbonaceous aerosols. De la Rosa et al. [35] determined the pyrogenic carbon in the surface sediments of the Guadiana River in the southwest of the Iberian Peninsula. The findings indicated that fossil fuel and C3 plant combustion were the main sources of pyrogenic carbon, while the black carbon produced by rock weathering sources was negligible.

Urban vegetation can filter pollutants to improve urban air quality, and green vegetation has always been considered a low-cost and effective method for passive monitoring of air pollution. Baldacchini et al. [36] sampled the PM particles deposited on the leaves of the *Platanus acerifolia* tree in 28 cities in 20 European countries and examined if the deposition particles on the leaf could be used as indicators of atmospheric PM concentration and composition. Currently, there are some large-scale experiments in continental Europe (e.g., the European Network for the Assessment of Air Quality by the Use of Bioindicator Plants Cooperative and European Survey of Atmospheric Heavy Metal Deposition) with leaves to measure the deposition of particulate matter and the metal composition of the ambient PM samples [37]. Rindy et al. [38] observed elemental carbon particles in PM on the leaves of oak trees in Texas, USA, and found that the oak canopy can accumulate 160–299 mg m^−2^ soot particles per year, and estimated that both oak species accumulate approximately 2.5 tons of elemental carbon particles per year.

Our prior research documented that the plant wax layer could adsorb soot particles and can be used as a biomonitoring tool to understand the major sources of atmospheric submicron soot in areas where air pollution measurements of soot emissions are absent [27]. However, our previous study is limited to one tree species (i.e., *Platanus acerifolia*). Since it is not documented whether the deposition of δ^13^C values in soot emission is associated with tree species, this study aims to test the hypothesis that the deposition of δ^13^C values is independent of tree species. The findings from this study could add practical value to the identification of soot sources using tree leaves as a biomonitoring tool.

## 2. Materials and Methods

### 2.1. Sample Collection

**Figure 1:**
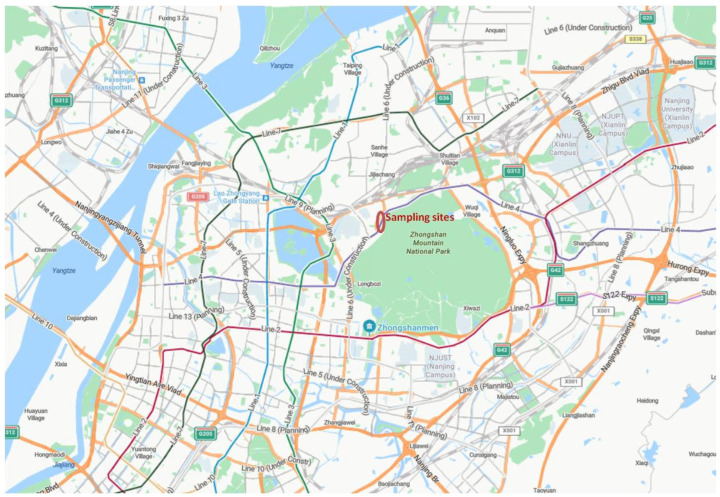
The locations of the sampling sites in Nanjing, China.

The sampling locations were situated in urban areas of Nanjing City, Jiangsu Province (Figure 1) [27]. Leaf samples were collected during four separate sampling periods in June (summer) and December (winter) of 2023, as well as June (summer) and December (winter) of 2024. The samples were taken from seven tree species following the National Atmospheric Deposition Programme (NADP) monitoring protocol [38]. The selected species included *Eucommia ulmoides*, *Koelreuteria paniculata*, *Osmanthus fragrans*, *Buddleja davidii*, *Platanus acerifolia*, *Fatsia japonica*, and *Hedera helix*. These seven plants (e.g., *Eucommia ulmoides*, *Koelreuteria paniculata*, *Osmanthus fragrans*, *Buddleja davidii*, *Platanus × acerifolia*, *Fatsia japonica*, and *Hedera helix*) are tolerant of pruning and well-suited for urban landscaping, making them common choices in gardens and parks [39,40]. They share some similarities in phyllotaxy, leaf margin, venation, and strong adaptability [39,40]. The leaves of *Osmanthus fragrans*, *Eucommia ulmoides*, *Fatsia japonica*, and *Hedera helix* are thick and tough, while those of *Koelreuteria paniculata*, *Buddleja davidii*, and *Platanus × acerifolia* are relatively thin yet still durable [39,40]. Based on prior studies, the wax content of their leaves varies between 1% and 3% of dry weight [39,40]. Each sample consisted of a composite of four leaves collected from a single representative tree. Trees of similar height (3–5 m) and trunk diameter (30–50 cm) were selected across all sampling areas in each season. Leaves were collected from the south-facing side of each tree (between 135 and 225 degrees), where black carbon emissions from mobile sources are most concentrated. Sampling was conducted at a naturally contaminated condition on non-rainy days to avoid wash-off of deposited black carbon or contamination from rainwater. During sample collection, we followed the National Atmospheric Deposition Programme (NADP) monitoring protocol to minimize the influence of precipitation and secondary deposition on leaf soot accumulation [41]. Mature leaves of similar age were collected three days after rainfall to ensure consistent deposition conditions. All sampled trees had been growing in the same location for at least three years, guaranteeing comparable exposure to deposition, meteorological factors, and emissions. Therefore, all collected leaves were subjected to identical weather conditions prior to sampling. In each season, six samples per tree species were collected. The same selection criteria were applied across all tree species. Approximately 20 g of leaves were collected per sample and a lot of 90 samples in one year were analyzed. After collection, the leaves were placed in pre-labeled plastic bags, stored in a portable refrigerator at 4 °C, and transported to the laboratory for further processing.

### 2.2. Sample Pretreatment

Before extraction, the leaf area was measured using a leaf area meter (Beijing Yaxin). Freshly collected intact leaves of seven species were used. The collected leaves of *Osmanthus fragrans*, *Eucommia ulmoides*, *Fatsia japonica*, and *Hedera helix* exhibit thick and tough textures, whereas those of *Koelreuteria paniculata*, *Buddleja davidii*, and *Platanus × acerifolia* are thinner but remain durable. The leaf wax content in the collected leaves ranges from 1% to 3% of dry weight [39,40]. Before the measurement, the leaf area meter is calibrated using the standard calibration plate from the instrument (Beijing Yaxin) [42]. Next, the collected leaves were each placed flatly on the scanning platform, and then leaf area data were obtained through scanning and image analysis. After the measurement was completed, the leaf area meter displayed the numerical value of the leaf area on the display screen.

The leaf samples were then extracted with a solution containing 20 mL of de-ionized water and 0.1 g of ammonium dihydrogen phosphate ((NH_4_)H_2_PO_4_). The extraction process was carried out three times on a centrifuge at 200 r min^−1^, with 15 min intervals between each cycle. The resulting aqueous extract was stored at 4 °C for 24 h. Prior to soot measurements, the extract was filtered through a 0.22 μm polytetrafluoroethylene (PTFE) syringe filter to remove micron-sized particles. Blank samples were prepared using quartz filters exposed to ambient outdoor air and processed following the same extraction protocol. One blank sample was collected for every 10 experimental samples to account for potential background contamination. To assess the reliability and accuracy of the developed method, spiking experiments were performed. When soot samples were spiked into leaf samples, the average recovery rates were found to range from 90% to 95%, with relative standard deviations below 5%. These results indicate that the method exhibits good precision and accuracy, ensuring its effectiveness for submicron soot determination in water extracts (Table S1).

### 2.3. Soot Measurements

The submicron soot content in the aqueous leaf extracts was quantified using a Multi N/C 3000 analyzer (Analytik Jena AG, Jena City, Germany) [27]. Before analysis, a blank sample (de-ionized water with 10% phosphoric acid) was introduced into the instrument. The sample extract, containing submicron soot and 10% H_3_PO_4_, was then mixed under a nitrogen atmosphere in the presence of a MnO_2_ catalyst and heated at 150 °C for two min in a block heater. During this digestion process, the soot was converted to CO_2_, which was then cooled in a condensing coil and separated from the condensate in a subsequent condensing tube. After additional drying and removal of corrosive gases, the CO_2_ concentration was measured using the analyzer’s non-dispersive infrared (NDIR) detector. Potential interference from organic carbon in the matrix was considered negligible, as the heating temperature under nitrogen was insufficient to convert organic carbon into CO_2_ (Table S2). The final concentration of soot in the leaf water extract was determined by subtracting the blank sample value from the measured sample value. Submicron soot accumulation (mg m^−2^) on the leaves was calculated by normalizing the soot concentration in the extract to the leaf surface area. It is noteworthy that several methods for soot determination (e.g., thermal-optical transmittance, an aethalometer, and visible spectrophotometry) are well-documented. The results obtained from our proposed method, using a Multi N/C 3000 analyzer, are consistent with those derived from ultraviolet–visible spectrophotometry, as recommended by the Chinese National Standards (GB 34323-2017) for black carbon determination (Figure S1 and Table S3) [43].

### 2.4. Stable Carbon Isotopes

In this study, δ^13^C values were determined in water extracts from collected leaf samples. The stable carbon isotope (δ^13^C) in leaf water extract was analyzed by a Thermo Scientific Delta V Plus (Thermo Scientific, Waltham City, MA, USA) [27]. Before measurement, the 12 mL sample bottle (Labco, London City, UK) was soaked in dilute hydrochloric acid (2 mM) for 48 h and then washed three times with ultra-pure water. Next, 1.5 mL water extracts for each sample were added into 2 mL 10% phosphoric acid and then kept in a heater at 45 °C for 45 min. After the heating, the water extract was centrifuged at 4000 r min^−1^ for 2 min and determined by isotope mass spectrometry under conditions of ultra-high purity helium (180 mL min^−1^). The samples combusted with oxygen were converted to CO_2_ and then analyzed with an isotope ratio mass spectrometer. The δ^13^C value was calibrated by the standard reference materials graphite (USGS 24, δ^13^C = −16.049‰) and sucrose (IAEA-CH-6, δ^13^C = −10.449‰). To check the stability of the instrument, the laboratory standard of graphite was analyzed every nine samples. The measurement error of δ^13^C is within 0.1‰.

The δ^13^C level in leaf samples was estimated using the following Equation (1):

(1) $$\delta^{13}C\left[‰\right]=\frac{{R(}^{13}C{/}^{12}C{)}_{\mathrm{sample}}}{{R(}^{13}C{/}^{12}C{)}_{\mathrm{std}}}$$

where R(^13^C/^12^C) sample and R(^13^C/^12^C)_std_ (= 0.1111802) refer to the ^13^C/^12^C ratio of the sample value and standard value (ViennaPee Dee Belemnite). Data measurements were repeated three times for all samples.

### 2.5. Isotope Mass Balance Model

We performed a simple binary mixture model to calculate the source contribution of biomass (f_biomass_) and fossil fuels (f_fossil_ = 1 − f_biomass_) to submicron soot on the leaf surface with Equation (2) [29]. The equation is shown as follows.

(2) $$\delta^{13}C_{\mathrm{aerosol}}=f_{\mathrm{biomass}\;}\times {\;\delta }^{13}C_{\mathrm{biomass}}+(1\;-{\;f}_{\mathrm{biomass}})\;\times \;\delta^{13}C_{\mathrm{fossil}}$$

where δ^13^C aerosol refers to the δ^13^C value of submicron level soot on the leaf surface, δ^13^C biomass is the δ^13^C value of biomass combustion (e.g., −34‰ to −25‰ for C3 plants, and −13‰ for C4 plants), and δ^13^C fossil represents the δ^13^C value of fossil fuel (e.g., −28‰ to −21‰) [27]. Because the sampling site is far away from the coastal areas, the contribution of marine pollution is not accounted for in the contribution. In this study, we chose the mean values of δ^13^C for coal combustion and liquid petroleum as the input of δ^13^C fossil fuel burning, and the mean values of δ^13^C for C3 and C4 plant combustion as the input of δ^13^C biomass burning [27]. In addition, sensitivity tests were conducted to validate the selected δ^13^C input values from emission sources (Table S4).

### 2.6. Statistical Analysis

The soot concentrations for four individual seasons were shown as means ± standard deviations (SDs). Analysis of variance (ANOVA) was used to examine the difference in submicron soot levels and δ^13^C values across different samples. All statistical analyses were performed using SPSS V20.0 with a significance level of 0.05.

## 3. Results and Discussion

### 3.1. Impacts of Carbonate Interference

To verify the effect of phosphate on eliminating the carbonate interference on the soot measurements in leaf samples, the contents of inorganic carbon in several blank reagents were investigated. As shown in Table 1, the levels of inorganic carbon were below the detection limits (0.02 μg mL^−1^) in de-ionized water and 2 mg mL^−1^ of NH_4_H_2_PO_4_. The concentration of inorganic carbon was not detected in a solution of 5 μg mL^−1^ carbonate and 2 mg mL^−1^ NH_4_H_2_PO_4_, which indicates that 2 mg mL^−1^ NH_4_H_2_PO_4_ could effectively remove the interference of carbonate for the determination of inorganic carbon in de-ionized water. Furthermore, three blank samples were chosen in the experiment to examine the effects of phosphate on minimizing the carbonate interference in real samples. The levels of inorganic carbon in the three blank samples were found to be 0.02 ± 0.01, 0.03 ± 0.01, and 0.03 ± 0.01 μg mL^−1^, respectively. Then, 5 μg mL^−1^ carbonate were added to the three blank samples. After the filtration and purification processes of blank samples, the concentration of inorganic carbon varied slightly compared to those in blank samples without the addition of 5 μg mL^−1^ carbonate. These results indicated that the interference of carbonate in a solution of 2 mg mL^−1^ NH_4_H_2_PO_4_ on the determination of submicron soot in leaf samples can be negligible.

### 3.2. Levels of Soot Across Species

**Figure 2:**
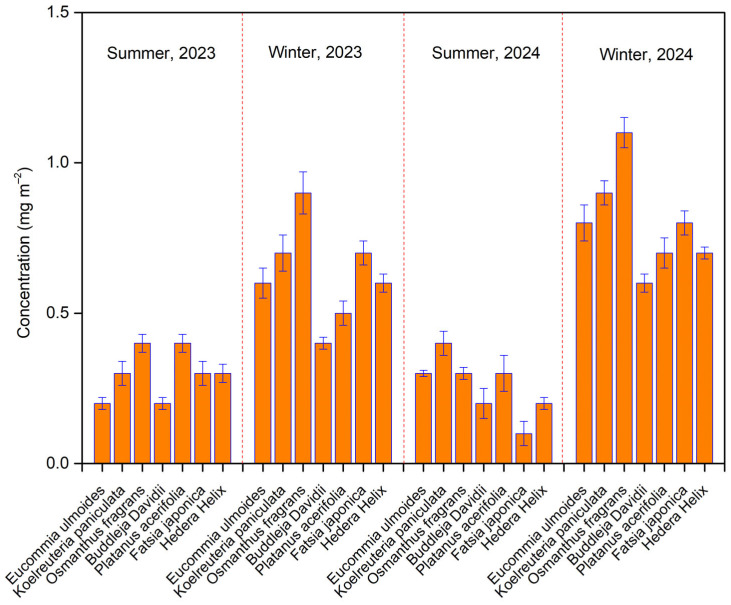
The concentrations of soot on leaves of seven tree species over four individual sampling periods.

Figure 2 shows that the soot concentrations on leaves from the seven tree species ranged from 0.2 to 1.3 mg m^−2^, consistent with previously reported submicron soot levels on *Platanus acerifolia* [27]. Average submicron soot concentrations were between 0.3 and 0.5 mg m^−2^ in summer, while winter levels were notably higher, ranging from 0.5 to 1.3 mg m^−2^. This study revealed significant variations in submicron soot accumulation among the seven species, even under similar environmental conditions. Usually, two factors limit the statistical power of ANOVA tests and increase the risk of both Type I (false positive) and Type II (false negative) errors [44]. In this study, the experimental design controlled for confounding factors (i.e., meteorological factors), thereby reducing the risk of Type I errors [44]. With sufficient sample size, the potential for Type II errors was minimized. Prior studies have indicated that the wax content of leaves plays a significant role in soot accumulation [44]. The wax contents of leaves in these seven species are reported to range from 1% to 3% of dry weight [39,40]. The significant interspecific variations in submicron soot accumulation can likely be attributed to differences in leaf wax content [39,40]. Additionally, leaf morphological characteristics (e.g., pubescent vs. glabrous surfaces) may influence particle adsorption and subsequent soot deposition [39,40]. These findings suggest that interspecific differences in soot accumulation are more strongly associated with leaf anatomical traits than with local emission levels. Notably, comparative analyses of foliar soot accumulation capacity should be conducted within the same species to ensure valid comparisons. Furthermore, all species exhibited substantially higher soot concentrations in winter than in summer. This seasonal increase may result from heightened biomass combustion for heating and elevated vehicle emissions during colder months [27,38]. Additionally, the lower atmospheric boundary layer in winter restricts vertical aerosol dispersion, worsening pollution accumulation [15,45]. In contrast, summer conditions—characterized by greater atmospheric turbulence and precipitation—enhance the diffusion and wet deposition of black carbon aerosols [16]. These findings demonstrate that tree leaves can serve as effective bioindicators of regional soot concentration fluctuations, reflecting seasonal changes in emission patterns and atmospheric conditions.

Since soot is a component of atmospheric aerosols, its accumulation on leaves serves as an indicator of ambient soot pollution levels [15]. The relationship between leaf-deposited soot and atmospheric concentrations helps reveal the deposition mechanisms of submicron soot particles onto vegetation. This transfer process depends on multiple environmental and meteorological factors (including rainfall, wind speed, and direction) as well as plant characteristics (such as leaf density, canopy structure, and tree height) [36,46]. While these factors are known to influence soot deposition, the current study’s scope does not allow for a comprehensive analysis of their specific impacts on submicron soot transfer from the atmosphere to leaf surfaces.

### 3.3. Sources of Soot

**Figure 3:**
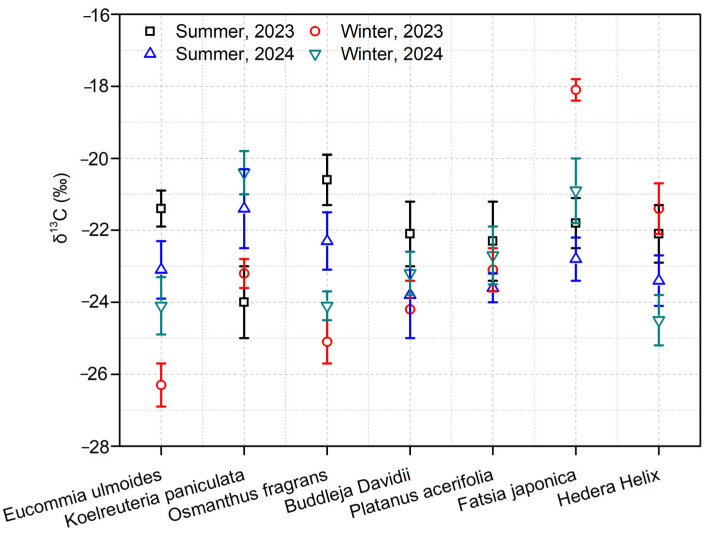
Trends in δ^13^C values on leaves of seven tree species over four individual sampling periods.

Stable carbon isotopes of soot aerosols extracted from the leaves of seven tree species at the sampling sites across four sampling periods were analyzed. The findings revealed that the overall variation range of δ^13^C values in submicron soot during winter was from −26.3‰ to −20.9‰, with an average of −24‰ ± 2‰. On the contrary, in summer, the overall variation of submicron soot δ^13^C ranged from −24.0‰ to −18.1‰, averaging −20.2‰ ± 1.1‰ (Figure 3). The differences in δ^13^C values of submicron soot among different species were relatively small. This suggests that the sampled leaves in this study can serve as excellent indicators for evaluating the source status of ambient submicron soot. The δ^13^C values of submicron soot from Nanjing were compared with the characteristics of carbon stable isotopes from various emission sources. In winter, the δ^13^C values of submicron soot predominantly fell within the range of fossil fuels, while some values were within the ranges of biomass burning sources and marine sources [30,34]. Given that the sampling sites were far from the ocean, the contribution of marine sources was ruled out. This indicates that fossil fuels made a substantial contribution to the soot levels in Nanjing during winter, and biomass burning also contributed to some degree. Similarly, in summer, the δ^13^C values of submicron soot mainly fell within the range of fossil fuels, with some values in the range of biomass burning. In summary, the soot levels on the leaves in Nanjing during both winter and summer were primarily influenced by the combustion of fossil fuels and biomass burning. The higher values in winter might be associated with the increased contribution of biomass burning.

**Figure 4:**
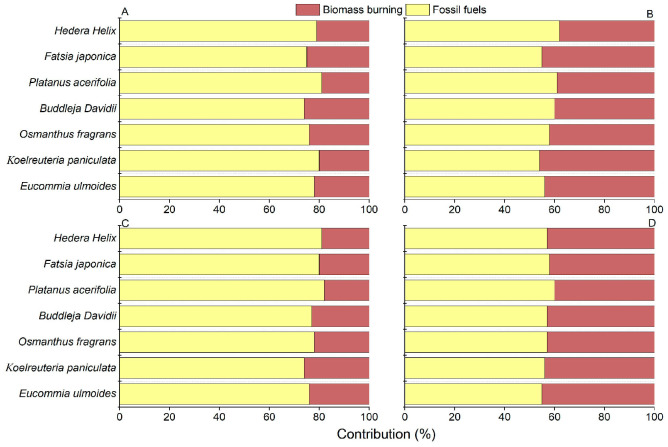
Relative contributions of fossil fuels and biomass burning to soot on leaves of seven tree species over four individual sampling periods. (**A**). Summer, 2023. (**B**). Winter, 2023. (**C**). Summer, 2024. (**D**). Winter, 2024.

The average contribution of biomass combustion is much greater in winter than that in summer. The average contribution of submicron soot in summer is higher from fossil fuels (78%), followed by biomass combustion (22%) (Figure 4). This result supports the previous research findings [27]. Winter submicron soot in the Nanjing area is primarily contributed by fossil fuels (56%), with a higher contribution from biomass combustion (44%). Biomass is an important energy source for the electric power sector, with common processes including large-scale biomass burning for energy supply and rural household stove burning. The results from this study provided evidence that the contribution of biomass burning to submicron soot on tree leaves was increased in winter compared to summer.

## 4. Conclusions

This study introduces a reliable method for quantifying sources of submicron soot levels on leaves of trees using a passive biomonitoring method. Our approach encourages additional exploration of submicron soot pollution and its associated emission sources, particularly by leveraging cost-effective biomonitoring techniques in developing regions grappling with substantial air pollution problems. Through the application of isotope mass balance calculations in conjunction with stable carbon isotope measurements, we discovered that during winter, submicron soot in Nanjing’s urban area primarily stems from fossil fuel emissions. The study’s findings suggest that the capacity of various tree species to adsorb soot aerosols differs considerably. However, the isotopic composition of dry deposition aerosols can be compared across different species. The results of this research underscore the potential of tree leaves as a biomonitoring tool for assessing the source status of submicron soot particles. These insights can aid developing countries with high levels of air pollution in monitoring submicron soot-related air pollution and estimating source contributions in an environmentally friendly manner by making use of street trees.

## Figures and Tables

**Table 1:** The concentrations of blank samples.

Samples	Concentration (μg mL^−1^)	δ^13^C(‰)
De-ionized water (n = 7)	N.D.	/
2 mg mL^−1^ NH_4_H_2_PO_4_ (n = 7)	N.D.	/
5 μg mL^−1^ carbonate and 2 mg mL^−1^ NH_4_H_2_PO_4_ (n = 7)	N.D.	/
Blank sample 1 and 2 mg mL^−1^ NH_4_H_2_PO_4_ (n = 7)	0.02 ± 0.01	−27.12
Blank sample 2 and 2 mg mL^−1^ NH_4_H_2_PO_4_ (n = 7)	0.03 ± 0.01	−26.89
Blank sample 3 and 2 mg mL^−1^ NH_4_H_2_PO_4_ (n = 7)	0.03 ± 0.01	−26.97

N.D.: not detectable; detection limits: 0.02 μg mL^−1^; n: number.

## Data Availability

The data are available in the main text.

## Supplementary Materials

The following supporting information can be downloaded at: https://www.mdpi.com/article/10.3390/biology14060583/s1, Figure S1. The standard curves for the determination of nanoscale graphene oxide (0.5–20 μg mL^−1^) and nanoscale carbon black (0.5–20 μg mL^−1^). Table S1. Sample recoveries of soot extraction. Table S2. Results from potential interference from water soluble organic carbon. Table S3. The concentration of soot determined by the Multi N/C analyzer and the ultraviolet—visible light absorbance. Table S4. δ13C values of soot from different emissions sources [27,29].

## References

[1] Chameides W.L., Bergin M. (2002). Soot Takes Center Stage. Science.

[2] Moffet R.C., Prather K.A. (2009). In-situ measurements of the mixing state and optical properties of soot with implications for radiative forcing estimates. Proc. Natl. Acad. Sci. USA.

[3] Rosenfeld D., Zhu Y., Wang M., Zheng Y., Goren T., Yu S. (2019). Aerosol-driven droplet concentrations dominate coverage and water of oceanic low-level clouds. Science.

[4] Andreae M.O., Ramanathan V. (2013). Climate’s Dark Forcings. Science.

[5] Sun X., Hu L., Hu B., Sun X., Wu X., Bi N., Lin T., Guo Z., Yang Z. (2022). Remarkable signals of the ancient Chinese civilization since the Early Bronze Age in the marine environment. Sci. Total Environ..

[6] Hansen J., Nazarenko L. (2004). Soot climate forcing via snow and ice albedos. Proc. Natl. Acad. Sci. USA.

[7] Gili J., Maín A., van Drooge B.L., Viana M. (2025). Source-resolved black carbon and PM2.5 exposures during wildfires and prescribed burns. Environ. Pollut..

[8] Thompson S.A., Aiken A.C., Huber R.C., Dubey M.K., Brooks S.D. (2024). Detonation Soot: A New Class of Ice Nucleating Particle. J. Geophys. Res. Atmos..

[9] Lill E., Costa E.J., Barry K., Mirrielees J.A., Mashkevich M., Wu J.D.Y., Holen A.L., Cesler-Maloney M., DeMott P.J., Perkins R. (2024). The Abundance and Sources of Ice Nucleating Particles Within Alaskan Ice Fog. J. Geophys. Res. Atmos..

[10] Testa B., Durdina L., Edebeli J., Spirig C., Kanji Z.A. (2024). Simulated contrail-processed aviation soot aerosols are poor ice-nucleating particles at cirrus temperatures. Atmos. Chem. Phys..

[11] Adams M.P., Tarn M.D., Sanchez-Marroquin A., Porter G.C.E., O’Sullivan D., Harrison A.D., Cui Z., Vergara-Temprado J., Carotenuto F., Holden M.A. (2020). A Major Combustion Aerosol Event Had a Negligible Impact on the Atmospheric Ice-Nucleating Particle Population. J. Geophys. Res. Atmos..

[12] Kärcher B., Kleine J., Sauer D., Voigt C. (2018). Contrail Formation: Analysis of Sublimation Mechanisms. Geophys. Res. Lett..

[13] Zhong J., Li Y.H., Bloss W.J., Harrison R.M. (2025). Street-scale black carbon modelling over the West Midlands, United Kingdom: Sensitivity test of traffic emission factor adjustments. Environ. Int..

[14] Yang Y., Ma M.H., Zhou L., Wang W.C., Li F.S. (2025). Study on the effect of soot generation from metal oxide/biodiesel nanofluid fuel combustion. Renew. Energy.

[15] Zhang Z.Q., Li D.M., Niu C.Y., Pan M.Z., Guan W., Liu H., Lu K., Tan D.L. (2024). Soot formation mechanism of modern automobile engines and methods of reducing soot emission for catalyzed diesel particulate filter: A review. Process Saf. Environ. Prot..

[16] Michelsen H.A., Colket M.B., Bengtsson P.E., D’Anna A., Desgroux P., Haynes B.S., Miller J.H., Nathan G.J., Pitsch H., Wang H. (2020). A Review of Terminology Used to Describe Soot Formation and Evolution under Combustion and Pyrolytic Conditions. ACS Nano.

[17] Liu H., Qi L.J., Liang C.S., Deng F.Y., Man H.Y., He K.B. (2020). How aging process changes characteristics of vehicle emissions? A review. Crit. Rev. Environ. Sci. Technol..

[18] Naidja L., Ali-Khodja H., Khardi S. (2018). Sources and levels of particulate matter in North African and Sub-Saharan cities: A literature review. Environ. Sci. Pollut. Res..

[19] Gao Y.Q., Ge Y.H., Ma Y.F., Zhao H.L., Xiao G.X., Show P.L., Chen J.Q., Guo R.X., Liu Y.H. (2023). Occurrence, Migration, and Transformation of Black Carbon in Environmental Matrix and Its Influence on the Environmental Fate of Coexisting Pollutants: A Review. Rev. Environ. Contam. Toxicol..

[20] Hu Z.F., Kang S.C., Li C.L., Zhang C., Yan F.P., Chen P.F., Danmuzhen D. (2024). Fifty percent overestimation of black carbon concentration measured in aerosols of the Tibetan Plateau. Environ. Pollut..

[21] Wang X.X., Luo X., Zhang Y.L., Kang S.C., Chen P.F., Niu H.W. (2024). Black carbon: A general review of its sources, analytical methods, and environmental effects in snow and ice in the Tibetan Plateau. Environ. Sci. Pollut. Res..

[22] Maricq M.M. (2023). Engine, aftertreatment, fuel quality and non-tailpipe achievements to lower gasoline vehicle PM emissions: Literature review and future prospects. Sci. Total Environ..

[23] Ramanathan V., Carmichael G. (2008). Global and regional climate changes due to black carbon. Nat. Geosci..

[24] Simoneit B.R.T. (2002). Biomass burning—A review of organic tracers for smoke from incomplete combustion. Appl. Geochem..

[25] Wang R., Tao S., Wang W.T., Liu J.F., Shen H.Z., Shen G.F., Wang B., Liu X.P., Li W., Huang Y. (2012). Black Carbon Emissions in China from 1949 to 2050. Environ. Sci. Technol..

[26] Li B.G., Gasser T., Ciais P., Piao S.L., Tao S., Balkanski Y., Hauglustaine D., Boisier J.P., Chen Z., Huang M.T. (2016). The contribution of China’s emissions to global climate forcing. Nature.

[27] Tao M.M., Liu Q.Y., Schauer J.J. (2022). Direct measurement of the deposition of submicron soot particles on leaves of *Platanus acerifolia* tree. Environ. Sci. Process. Impacts.

[28] Bird M.I., Ascough P.L. (2012). Isotopes in pyrogenic carbon: A review. Org. Geochem..

[29] Zhan C.L., Wan D.J., Zhang J.Q., Han Y.M., Cao J.J., Liu X.L. (2016). Research progress on source apportionment methods of black carbon in the environment. Ecol. Environ. Sci..

[30] Suto N., Kawashima H. (2021). Measurement report: Source characteristics of water-soluble organic carbon in PM_2.5_ at two sites in Japan, as assessed by long-term observation and stable carbon isotope ratio. Atmos. Chem. Phys..

[31] Winiger P., Andersson A., Yttri K.E., Tunved P., Gustafsson Ö. (2015). Isotope-Based Source Apportionment of EC Aerosol Particles during Winter High-Pollution Events at the Zeppelin Observatory, Svalbard. Environ. Sci. Technol..

[32] Wynn J.G., Bird M.I. (2007). C4-derived soil organic carbon decomposes faster than its C3 counterpart in mixed C3/C4 soils. Glob. Change Biol..

[33] Wynn J.G., Bird M.I. (2008). Environmental controls on the stable carbon isotopic composition of soil organic carbon: Implications for modelling the distribution of C_3_ and C_4_ plants, Australia. Tellus B Chem. Phys. Meteorol..

[34] Liu X.Q., Wang Z.D., Wang J.Z., Xing L., Li J.Y., Dong Z.B., Li M.R., Han Y.M., Cao J.J. (2024). Characteristics of PM 2.5 bounded carbonaceous aerosols, carbon dioxide and its stable carbon isotopes (δ 13 C) in rural households in northwest China: Effect of different fuel combustion. J. Environ. Manag..

[35] De la Rosa J.M., Martins J.M., Soares A.M., Araújo M.F. (2015). Assessment of distribution and sources of pyrogenic carbon in the lower course of the Guadiana River (SW Iberian Peninsula). J. Soils Sediments.

[36] Baldacchini C., Castanheiro A., Maghakyan N., Sgrigna G., Verhelst J., Alonso R., Amorim J.H., Bellan P., Bojovic D.D., Breuste J. (2017). How Does the Amount and Composition of PM Deposited on *Platanus acerifolia* Leaves Change Across Different Cities in Europe?. Environ. Sci. Technol..

[37] Klumpp A., Ansel W., Klumpp G., Belluzzo N., Calatayud V., Chaplin N., Garrec J.P., Gutsche H.J., Hayes M., Hentze H.W. (2002). EuroBionet: A Pan-European biomonitoring network for urban air quality assessment. Environ. Sci. Pollut. Res..

[38] Rindy J.E., Ponette-González A.G., Barrett T.E., Sheesley R.J., Weathers K.C. (2019). Urban Trees Are Sinks for Soot: Elemental Carbon Accumulation by Two Widespread Oak Species. Environ. Sci. Technol..

[39] Afzal S., Singh N.K., Lal A.F., Sohrab S., Singh N., Gupta P.S., Mishra S.K., Adeel M., Faizan M. (2024). Nanostructure and plant uptake: Assessing the ecological footprint and root-to-leaf dynamics. Plant Nano Biol..

[40] George R., Thuseethan S., Ragel R.G., Mahendrakumaran K., Nimishan S., Wimalasooriya C., Alazab M. (2025). Past, present and future of deep plant leaf disease recognition: A survey. Comput. Electron. Agric..

[41] Fernández J.A., Boquete M.T., Carballeira A., Aboal J.R. (2015). A critical review of protocols for moss biomonitoring of atmospheric deposition: Sampling and sample preparation. Sci. Total Environ..

[42] Yan G., Hu R., Luo J., Weiss M., Jiang H., Mu X., Xie D., Zhang W. (2019). Review of indirect optical measurements of leaf area index: Recent advances, challenges, and perspectives. Agric. For. Meteorol..

[43] (2017). Carbon Black—Determination of Light Transmittance of Water Dispersion—Spectrophotometer Method.

[44] Hussey I. (2023). A systematic review of null hypothesis significance testing, sample sizes, and statistical power in research using the Implicit Relational Assessment Procedure. J. Context. Behav. Sci..

[45] Tiwari S., Pandithurai G., Attri S.D., Srivastava A.K., Soni V.K., Bisht D.S., Kumar V.A., Srivastava M.K. (2015). Aerosol optical properties and their relationship with meteorological parameters during wintertime in Delhi, India. Atmos. Res..

[46] Pace R., Guidolotti G., Baldacchini C., Pallozzi E., Grote R., Nowak D.J., Calfapietra C. (2021). Comparing i-Tree Eco Estimates of Particulate Matter Deposition with Leaf and Canopy Measurements in an Urban Mediterranean Holm Oak Forest. Environ. Sci. Technol..

