# Supplementary material for: Sources of the Deposition of Submicron Soot Particles on Plant Leaves
Source: Biology (Basel). 2025 May 22;14(6):583. doi: 10.3390/biology14060583 (PMC12190160; doi:10.3390/biology14060583)
Supplement: Supplementary file 1 [file biology-14-00583-s001.zip › biology-3616585-supplementary.pdf]

Supplementary Materials

1. Sample recoveries of soot extraction method

2 μg mL^{-1} of nanoscale graphene oxide and nanoscale carbon black were added to the water extracts of leaf samples. Then, the leaf samples were combined with 2 mg mL^{-1} ammonium dihydrogen phosphate ($(NH_4)_2H_2PO_4$). The mixture was agitated on a rotation table set at 200 r min^{-1} for three consecutive 15-minute intervals. Subsequently, the solution was introduced into a Multi N/C analyzer (Analytik Jena AG, Germany) to measure inorganic carbon (IC). This experiment was replicated with seven parallel samples (Table S1). The resulting mixtures were then analyzed for IC using the Multi N/C analyzer. The recovery rates of these analytes were found to be in the range of 90 - 95%. Consequently, it can be concluded that the combination of water and $(NH_4)_2H_2PO_4$ could effectively extract the soot samples on leaf samples.

Table S1. Sample recoveries of soot extraction

Identifier	C Content (μg mL^{-1})	13δC(‰)
Milli-Q water (n=7)	ND	/
Milli-Q water and 2 mg mL^{-1} ammonium dihydrogen phosphate ($(NH_4)_2H_2PO_4$) (n=7)	ND	/
5 μg mL^{-1} carbonate in Milli-Q water and 2 mg mL^{-1} ammoniumdihydrogen phosphate ($(NH_4)_2H_2PO_4$) (n=7)	ND	/
2 μg mL^{-1} of nanoscale graphene oxide and nanoscale carbon black of leaf samples (Sample 1, n=7)	1.90±0.03	-27.63
2 μg mL^{-1} of nanoscale graphene oxide and nanoscale carbon black of leaf samples (Sample 2, n=7)	1.83±0.04	-27.41
2 μg mL^{-1} of nanoscale graphene oxide and nanoscale carbon black of leaf samples (Sample 3, n=7)	1.86±0.03	-27.14

ND: lower than the detection limit (0.02 μg mL^{-1})

2. The effects of water-soluble carbon on soot determination

Standard solutions were prepared by dissolving water-soluble brown carbon at concentrations ranging at 1, 5 and 10 μg mL^{-1}, respectively. The water-soluble brown carbon consisted of humic acids and a mixture of amino acids, including glycine, alanine, valine, leucine, isoleucine, proline, aspartate, glutamate, serine, and threonine. Subsequently, using a a Multi N/C analyzer, the solution was characterized with 10% H_3PO_4 under a nitrogen atmosphere in the presence of a MnO_2 catalyst and heated at 150°C for two minutes in a block heater. The experiments were conducted in seven parallel replicates (Table S2). In all samples, the levels of inorganic carbon were below the instrumental detection limit (0.02 μg mL^{-1}). Given these results, potential interference from water soluble organic carbon in submicron soot determination from leaf samples using the described measurement methods is negligible.

Table S2. Results from potential interference from water soluble organic carbon

Identifier	C Content (μg mL^{-1})
Milli-Q water (n=7)	ND
Milli-Q water and 2 mg mL^{-1} ammonium dihydrogen phosphate ($(NH_4)_2H_2PO_4$) (n=7)	ND
5 μg mL^{-1} carbonate in Milli-Q water and 2 mg mL^{-1} ammoniumdihydrogen phosphate ($(NH_4)_2H_2PO_4$) (n=7)	ND
1 μg mL^{-1} of water-soluble brown carbon and 2 mg mL^{-1} ammoniumdihydrogen phosphate ($(NH_4)_2H_2PO_4$) (n=7)	ND
5 μg mL^{-1} of water-soluble brown carbon and 2 mg mL^{-1} ammoniumdihydrogen phosphate ($(NH_4)_2H_2PO_4$) (n=7)	ND
10 μg mL^{-1} of water-soluble brown carbon and 2 mg mL^{-1} ammoniumdihydrogen phosphate ($(NH_4)_2H_2PO_4$) (n=7)	ND

ND: lower than the detection limit (0.02 μg mL^{-1})

3. Validation of submicron soot measurement

To validate the results determined by a Multi N/C analyzer, the determination of soot in water extracts adhered to the Chinese national standard method (GB34323 - 2017). Initially, standard solutions were formulated with nanoscale carbon black at concentrations ranging from 0.5 to 200 μg mL^{-1}. The nanoscale carbon black, with a diameter of 3 - 5 nm and of N234 grade, was commercially sourced from Alfa Aesar Chemical. (Haverhill, MA, USA), commonly utilized as an additive in rubber tires. Subsequently, the ultraviolet - visible light absorbance of submicron BC was measured using a Perkin - Elmer Lambda 950 spectrophotometer (Waltham, MA, USA). The absorbance was recorded at 0.1 nm intervals from 400 nm to 410 nm. To mitigate background interferences, a dual - wavelength ultraviolet - visible method was adopted. The differences in ultraviolet - visible absorption spectra at 405 nm and 410 nm were plotted against the submicron BC levels in the extraction solution (Figure S1). Under optimal conditions, the coefficient of determination for BC was over 0.99, with a linear range spanning from 0.5 to 200 μg mL^{-1}. The measured relative standard deviations (RSDs) fluctuated between 1.8% and 5.0%, and the detection limit was established at 0.1 μg mL^{-1}. Then, the concentration of soot in leaf samples were determined by the Multi N/C analyzer and the ultraviolet - visible light absorbance, respectively. The results obtained from our proposed method using a Multi N/C 3000 analyzer are consistent with those derived from visible spectrophotometry, as recommended by Chinese National Standards (GB 34323-2017) for black carbon determination (Table S3).

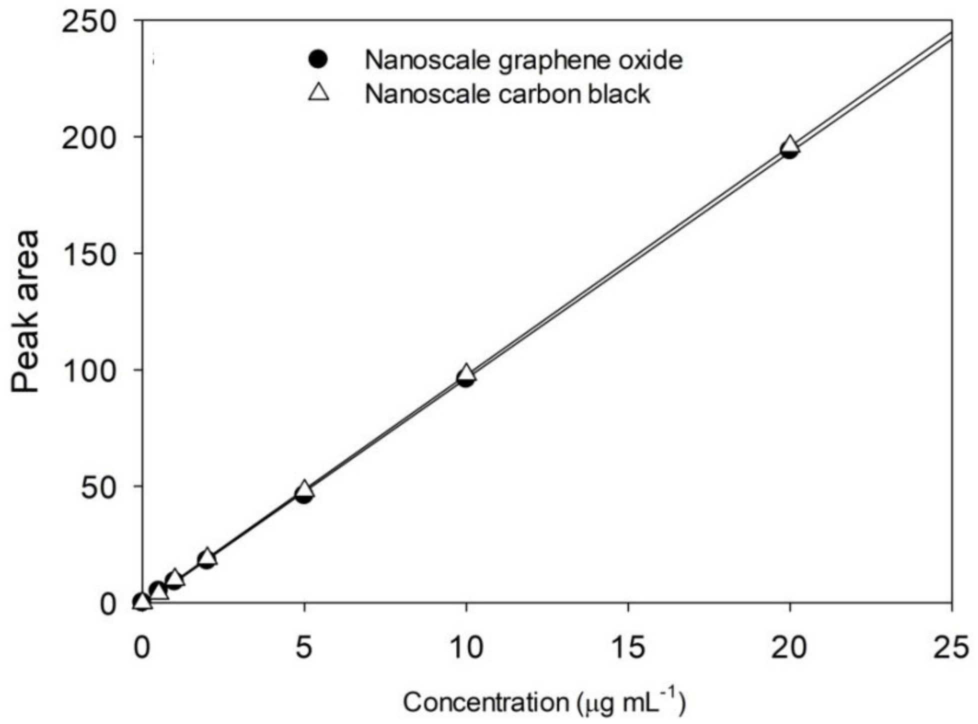

Figure S1. The standard curves for the determination of nanoscale graphene oxide (0.5-20 μg mL^{-1}) and nanoscale carbon black (0.5-20 μg mL^{-1}).

Table S3. The concentration of soot determined by the Multi N/C analyzer and the ultraviolet - visible light absorbance

Samples	Multi N/C analyzer (μg mL^{-1})	Ultraviolet - visible light absorbance (μg mL^{-1})
Sample 1 (n=7)	1.5±0.2	1.3±0.3
Sample 2 (n=7)	1.9±0.2	1.7±0.2
Sample 3 (n=7)	2.8±0.2	2.6±0.3

4. Sensitivity test for source apportionment using a isotope mass balance model

The $\delta^{13}C$ values of soot from various emission sources were derived from previous studies. Given that the $\delta^{13}C$ signatures of liquid petroleum closely resembled those of coal combustion in China, we combined their mean values to represent the $\delta^{13}C$ signature of fossil fuels. To assess source contributions to soot, we conducted sensitivity tests using an isotope mass balance model with two input scenarios: (1) the mean $\delta^{13}C$ value of liquid petroleum alone (Test I) and (2) the combined mean$\delta^{13}C$ values of coal combustion and liquid petroleum (Test II). The relative contribution of biomass burning to soot remained consistent between these tests because the$\delta^{13}C$ values of liquid petroleum and the combined fossil fuel sources were nearly identical.

Table S4. δ13C values of soot from different emissions sources [1, 2]

Emissions sources	$\delta^{13}C$/‰
Pine	-24.14
Diesel engined car-I	(-25.10 ± 0.42)
Diesel engined car-II	-25.3 -24.7
Diesel engined car-III (Summer)	(-24.4± 0.4)
Diesel engined car-III (Winter)	(-24.4± 0.2)
Diesel engined car-V	-24.9
Gasoline vehicle-I	-25.45
Gasoline vehicle-II	-23.6 -22.2
Gasoline vehicle-III	(-24.4± 0.7)
Passenger cars-I (Gasoline)	(-24.4± 0.7)
Passenger cars-II (Gasoline)	(-20.6 ± 1.5)
Bituminous coal	(-23.46 ± 0.37)
Fireplace ash	(-26.5 ± 0.1)
Charcoal	(-27.4 ± 1.7)

References

1. Zhan, C.L.; Wan, D.J.; Zhang, J.Q.; et al. Research progress on source apportionment methods of black carbon in the environment. Ecology and Environmental Sciences, 2016, 25(09): 1575–1583.
2. Tao, M.; Liu, Q.; Schauer, J.J. Direct measurement of the deposition of submicron soot particles on leaves of Platanus acerifolia tree. Environmental Science: Processes & Impacts, 2022, 24(12): 2336–2344.
